# Critically ill SARS-CoV-2-infected patients are not stratified as sepsis by the qSOFA

**DOI:** 10.1186/s13613-020-00664-w

**Published:** 2020-04-19

**Authors:** Marion Ferreira, Timothee Blin, Nived Collercandy, Piotr Szychowiak, Pierre-François Dequin, Youenn Jouan, Antoine Guillon

**Affiliations:** 1 CHRU de Tours, Service de Médecine Intensive-Réanimation, CHRU Bretonneau, INSERM U1100, Centre d’Etude des Pathologies Respiratoires, Université de Tours, 2 Bd Tonnellé, 37044 Tours, France; 2 CHRU de Tours, Service de Pneumologie, INSERM U1100, Centre d’Etude des Pathologies Respiratoires, Université de Tours, 2 Bd Tonnellé, 37044 Tours, France

The SEPSIS-3 definitions had facilitated earlier recognition of patients at risk of developing sepsis for timely management [1]. The quickSOFA (qSOFA) has emerged as a bedside clinical score to clinically categorize a septic patient. In out-of-hospital, emergency department, or general hospital ward settings, adult patients with suspected infection are likely to have poor outcomes typical of sepsis if they have at least 2 of the qSOFA criteria: respiratory rate ≥ 22/min, altered mentation, or systolic blood pressure ≤ 100 mmHg [1]. This definition has been subsequently validated in the emergency department for patients with suspected infection: for qSOFA ≤ 1 the mortality rate was 3% (95% CI 2–5%) *vs* 24% (95% CI 18–30%) for patients with a qSOFA ≥ 2 [2]. The severe acute respiratory syndrome coronavirus-2 (SARS-CoV-2) that causes coronavirus disease 2019 (Covid-19) is a game changer. Mass ICU care and ventilatory support are needed to treat patients with Covid-19. Prompt and accurate clinical identification of SARS-CoV-2-infected patients at risk to have poor outcomes is an utmost priority. The aim of the study was to examine if the 2-point qSOFA threshold is an appropriate bedside clinical score for Covid-19 patients.

We studied patients with laboratory-confirmed Covid-19 infection who were admitted to ICU between March 14 and April 03, 2020. We defined a confirmed case of Covid-19 by a positive result on a reverse-transcriptase–polymerase-chain-reaction assay of a specimen collected on a nasopharyngeal swab or collection of nasopharyngeal aspirate. We recorded anonymized demographic data, information on clinical symptoms or signs at presentation at emergency department, and laboratory and radiologic results during ICU admission (performed at the discretion of the treating physician). The qSOFA was calculated based on its 3 components at their worst level before ICU admission. The reuse of already recorded and anonymized data falls within the scope of the French Reference Methodology MR-005 (2016–41 law) which require neither information nor non-opposition of the included individuals. Results are in median (± SD).

We identified 52 critically ill patients hospitalized in ICU with confirmed SARS-CoV-2 infection. The median age of the patients was 63 ± 11 years (range 28 to 78); 61.5% were men. The median duration of symptoms before hospital admission was 8 ± 3.5 days. Thirty-eight patients (73%) received invasive mechanical ventilation; 14 patients (27%) were discharged without the need of ventilator support (median oxygen low rate 3.5 ± 3.6 L/min). For patients who received mechanical ventilation, the mean Pao2:Fio2 ratio was 146 ± 94: 6 patients (16%) had mild ARDS, 23 patients (61%) had moderate ARDS and 9 patients (24%) had severe ARDS [3]. Twenty-six patients (68%) were placed in a prone position, 36 (94%) received neuromuscular blockade. Twenty-one patients (55%) presented hypotension requiring vasopressors; three patients needed renal-replacement therapy. The qSOFA of non-ventilated patients was one or less for all the patients (n = 14) (Fig. 1a). The qSOFA of ventilated patients was one or less for 27 patients (87%), only 4 patients had a 2-point qSOFA, none had 3-point (Fig. 1b). All patients with qSOFA ≥ 1 scored for respiratory rate ≥ 22/min; patients with qSOFA = 2 scored also for systolic blood pressure ≤ 100 mmHg. Seven patients had missing values to calculate the qSOFA. The case fatality rate could not be calculated as 35 patients were still hospitalized in the ICU while writing this report.

**Fig. 1:**
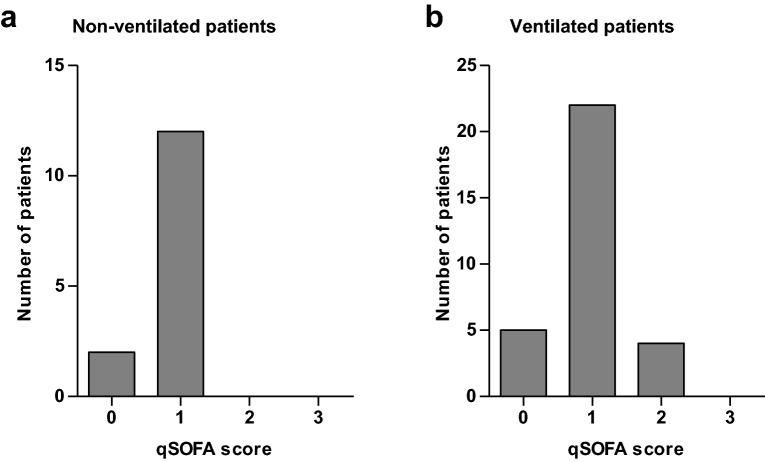
Distribution of patients by qSOFA score on ICU admission among confirmed Covid-19 patients. **a** Non-ventilated patients. **b** Ventilated patients

Covid-19 patients developing ARDS have a mean qSOFA score of one on ICU admission. Patients who received mechanical ventilation did not seem to have a different qSOFA than patients without ventilator support. A case fatality rate of 50% is expected among critically ill Covid-19 patients [4]. Consequently, the previous 3% mortality rate observed in patients with suspected infection and a qSOFA score ≤ 1 is unlikely to be exact in SARS-CoV-2-infected patients [1]. We anticipate that the qSOFA is not appropriate to identify Covid-19 patient to have poor outcomes typical of sepsis.

## Data Availability

Data are available from the authors upon reasonable request and with the permission of the institution.

## Abbreviations

ICU: Intensive care unit
qSOFA: quickSOFA
SARS-CoV-2: Severe acute respiratory syndrome coronavirus-2

## References

[1] Singer M, Deutschman CS, Seymour CW (2016). The third international consensus definitions for sepsis and septic shock (Sepsis-3). JAMA.

[2] Freund Y, Lemachatti N, Krastinova E (2017). Prognostic accuracy of sepsis-3 criteria for in-hospital mortality among patients with suspected infection presenting to the emergency department. JAMA.

[3] Definition Task Force ARDS, Ranieri VM, Rubenfeld GD (2012). Acute respiratory distress syndrome: the Berlin definition. JAMA.

[4] Bhatraju PK, Ghassemieh BJ, Nichols M (2020). Covid-19 in critically ill patients in the Seattle region—case series. N Engl J Med.

